# Oral cancer incidence and survival rates in the Republic of Ireland, 1994-2009

**DOI:** 10.1186/s12885-016-2839-3

**Published:** 2016-12-20

**Authors:** Hala Ali, Sarah-Jo Sinnott, Paul Corcoran, Sandra Deady, Linda Sharp, Zubair Kabir

**Affiliations:** 1 Department of Epidemiology and Public Health, University College Cork, Cork, Ireland; 2 Department of non-communicable disease epidemiology, London School of Hygiene and Tropical Medicine, Kepple St, London, WC1E 7HT UK; 3 Department of Obstetrics and Gynaecology, Cork University Maternity Hospital, Wilton, Cork, Ireland; 4 National Cancer Registry, Cork Airport Business Park, Kinsale Road, Cork, Ireland; 5 Institute of Health and Society, Newcastle University, The Baddiley-Clark Building, Richardson Road, Newcastle upon Tyne, NE2 4AX UK

**Keywords:** Oral cancer, Incidence rate, Survival rate, Joinpoint regression, Time trend

## Abstract

**Background:**

Oral cancer is a significant public health problem world-wide and exerts high economic, social, psychological, and physical burdens on patients, their families, and on their primary care providers. We set out to describe the changing trends in incidence and survival rates of oral cancer in Ireland between 1994 and 2009.

**Methods:**

National data on incident oral cancers [ICD 10 codes C01-C06] were obtained from the National Cancer Registry Ireland from 1994 to 2009. We estimated annual percentage change (APC) in oral cancer incidence during 1994–2009 using joinpoint regression software (version 4.2.0.2). The lifetime risk of oral cancer to age 79 was estimated using Irish incidence and population data from 2007 to 2009. Survival rates were also examined using Kaplan-Meier curves and Cox proportional hazard models to explore the influence of several demographic/lifestyle covariates with follow-up to end 2012.

**Results:**

Data were obtained on 2,147 oral cancer incident cases. Men accounted for two-thirds of oral cancer cases (*n* = 1,430). Annual rates in men decreased significantly during 1994–2001 (APC = -4.8 %, 95 % CI: −8.7 to −0.7) and then increased moderately (APC = 2.3 %, 95 % CI: −0.9 to 5.6). In contrast, annual incidence increased significantly in women throughout the study period (APC = 3.2 %, 95 % CI: 1.9 to 4.6). There was an elevated risk of death among oral cancer patients who were: older than 60 years of age; smokers; unemployed or retired; those living in the most deprived areas; and those whose tumour was sited in the base of the tongue. Being married and diagnosed in more recent years were associated with reduced risk of death.

**Conclusion:**

Oral cancer increased significantly in both sexes between 1999 and 2009 in Ireland. Our analyses demonstrate the influence of measured factors such as smoking, time of diagnosis and age on observed trends. Unmeasured factors such as alcohol use, HPV and dietary factors may also be contributing to increased trends. Several of these are modifiable risk factors which are crucial for informing public health policies, and thus more research is needed*.*

## Background

Oral cancer (OC) is a common cancer worldwide with an incidence of 300,000 cases in 2012, amounting for over 2 % of the overall burden of cancer diagnoses globally [1]. It is the 7th leading cause of death from cancer in Europe [2]. In Ireland, about 233 oral cancer cases are diagnosed annually [3] and over the last 5 years (2011–2015 inclusive), an average number of 71 deaths from oral cancers have been recorded [4]. In recent years, these numbers have been increasing [5]. A second issue of concern is the rising incidence of OC in women, which rose from 24 % in 1994 to 32 % in 2009 [5]. This is notable given that OC is traditionally more common in men [6].

Drinking alcohol alone is not an independent risk factor for OC but may have a mediating effect with smoking status [7, 8]. Although alcohol consumption in Ireland has decreased over the past ten years, it remained the highest amongst OECD (Organisation for Economic Co-operation and Development) countries in 2012 with a consumption of 11.6 L of alcohol per adult per year, in comparison to the OECD average of 9 l [9]. In 2010, Irish adults were still drinking more than twice the average amount of alcohol consumed per adult in 1960 [10]. This is accompanied by a culture of binge drinking which is not as problematic in other European countries [10].

However, significant improvements have been made in smoking behaviours which can be largely attributed to Ireland’s innovation in implementing a smoking ban in public places in 2004. The prevalence of smoking was 19.5 % in 2014 [11] compared to 29 % in 2007 [12]. Nevertheless, smoking remains high among the 25–34 year age group (27.3 %) and in the lower socioeconomic groups [11]. A third important risk factor for certain types of OC is Human papillomavirus (HPV), transmitted through conventional and oral sexual contacts [13]. It is a common infection, 70–80 % of Irish women will be infected with HPV at some stage in their life [14], although most will have the strength of immunity to clear the virus themselves [14]. In a study of 996 Irish women undergoing opportunistic cervical screening, overall HPV prevalence was 19.8 % [15]. Given recent advances in the development and mass administration of HPV vaccines, this is now a modifiable risk factor with potential to reduce the incidence of these cancers [13].

Other potential risk factors commonly discussed are radiation, over exposure to ultraviolet sunlight (involved in lip cancer) [5], genetic predisposition [16] and socioeconomic status which has been found to be significantly linked with increased oral tumour risk in both low and high income countries [17]. Heavy metals like nickel and chromium and poor oral hygiene may also play roles in OC etiology [18].

### Rationale for the study

Despite declines in mortality rates associated with OC in Ireland, recent evidence has pointed to an increase in incidence amongst certain demographic groups [19]. The changing profile of risk factors may have an explanatory role in this instance. Thus, in this study we aimed to first describe the changing trends of incidence of OC over the period 1994–2009. Second, we aimed to calculate the lifetime cumulative incidence risk of OC because such an estimate has not been undertaken in Ireland in recent years. Thirdly, we used relevant socio-demographic and clinical factors to explore survival rates in OC and to identify specific populations that may be at greater risk of mortality from oral cancers.

## Methods

This study was approved by the Clinical Research Ethics Committee of the Cork Teaching Hospitals, Ireland.

### Data

An anonymized dataset of oral cancer cases was obtained from the National Cancer Registry in Ireland (NCRI). The NCRI records all cancers newly diagnosed in the population resident in Ireland. Completeness of registration is estimated to be at least 97 % [20]. Based on the International Classification of Disease (ICD) tool from the World Health Organization oral cancer records were abstracted for cancers at the following sites; base of the tongue (C01), tongue (C02), gum (C03), floor of mouth (C04), palate (C05), and unspecified mouth (C06) for all patients aged over 15 years from 1994 up until the end of 2009. The NCRI follows cases by obtaining death certificates and linking these to registrations. Follow-up was complete to end 2012 [21].

### Covariates

We included several covariates in our analyses based on highlighted risk factors for OC in the international literature [6]; sex, age-group, marital status, and year of diagnosis. Further covariates included smoking status at diagnosis (current smokers, never smokers, ex-smokers, unknown) [22], occupation status and local area socio-economic status. Socioeconomic status was allocated to each record based on the electoral division (ED) of residence at the time of the patient’s diagnosis. This area-based index was developed by the Small Area Health Research Unit at Trinity College Dublin and is described in Williams et al. 2003 [23]. Each ED was assigned a deprivation index, which used five census-based indicators from the 2002 Irish census (representing the midpoint of the period included in this analysis): unemployment, low social class, car ownership, rented accommodation and overcrowding [24]. The deprivation index ranged from 1 (least deprived) to 5 (most deprived).

For the statistical analysis we collapsed the deprivation index 1, 2, 3 for least and 4, 5 for the most deprived score. We also included data on tumour site and tumour stage at diagnosis, which is a summary overall cancer stage based on the fifth edition of the American Joint Committee on Cancer (AJCC) TNM staging manual (1997) [25].

### Annual percent change (APC) 1994–2009

Incidence rates for oral cancer of Irish individuals were calculated using the joinpoint regression program (version 4.2.0.2) [26], which models the natural logarithm of the rates, identifying years at which any given trend changes, connecting these years graphically by a series of straight line segments [27]. They are expressed as the annual percent (APC) over the reported trend period. Incidence rates were age-adjusted and reported per 100, 000 population using the direct approach to the European age-standard population.

### Lifetime cumulative risk incidence

Lifetime cumulative risks of OC incidence were calculated using denominator data for the population at risk from the Central Statistics Office Ireland from 2007 to 2009 and numerator data from the NCRI database was the number of diagnosed cases in 5 year age groups starting at 15 years up to 79 (15-19, 20-24, 25-29, 30-34, 35-39, 40-44, 45-49, 50-54, 55-59, 60-64, 65-69, 70-74, 75-79) for the years 2007–2009 for both sexes.

Calculations were based on data aggregated over three years because oral cancer is relatively rare and the cumulative risk fluctuates from one year to the next.

### Survival analysis

We assessed the probability of cancer-specific survival using Kaplan-Meier (KM) curves. To examine the influence of lifestyle and clinical risk factors on survival probability we used a Cox regression assuming proportional hazards. Multivariable Cox regression models included age, sex, smoking, marital status, deprivation level, occupation, cancer site and cancer stage.

Results were reported as hazard ratios (HR) with equivalent 95 % confidence intervals (CI). All *p*-values were two tailed. The level of significance was set at 0.05. Data analysis was conducted using STATA (version 13.0).

## Results

Population-based data from the NCRI indicated that 2,147 individuals aged ≥15 years were diagnosed as new oral cancer cases between 1994 and 2009. Characteristics of the cases across three distinct periods (1994–1999, 2000–2004, 2005–2009) are presented below (Table 1). Of note, most cases occurred in men, in those aged over 60 years (median age of diagnosis was 63 years), in current smokers and in people who were most deprived.

**Table 1:** Characteristics of the 2,147 oral cancer patients diagnosed in 1994–2009

Years	1994–1999 (*n* = 728)	2000–2004 (*n* = 614)	2005–2009 (*n* = 805)	Total (*n* = 2147)
Women *n* (%)	214 (29.4)	211 (34.4)	292 (36.3)	717 (33.4)
Men *n* (%)	514 (70.6)	403 (65.6)	513 (63.7)	1430 (66.6)
Age (years) *n* (%)				
<30	6 (0.8)	7 (1.1)	19 (2.4)	32 (1.5)
30–60	224 (33.5)	262 (42.7)	309 (38.4)	815 (37.9)
> 60	478 (65.7)	345 (56.2)	477 (59.3)	1300 (60.6)
Married *n* (%)	353 (48.4)	317 (51.6)	422 (52.4)	1092 (50.9)
Divorced *n* (%)	5 (0.7)	10 (1.6)	14 (1.7)	29 (1.4)
Single *n* (%)	182 (25.0)	129 (21.0)	179 (22.2)	490 (22.8)
Other *n* (%)	188 (25.8)	158 (25.7)	190 (23.6)	536 (24.9)
Current smoker *n* (%)	414 (56.9)	297 (48.4)	385 (47.8)	1096 (51.1)
Non-smoker *n* (%)	131 (17.9)	142 (23.1)	180 (22.4)	453 (21.1)
Former smoker *n* (%)	91 (12.5)	107 (17.4)	128 (15.9)	26 (15.1)
Unknown *n* (%)	92 (12.6)	68 (11.1)	112 (13.9)	272 (12.7)
Employed *n* (%)	93 (12.7)	125 (20.4)	164 (20.3)	382 (17.8)
Unemployed *n* (%)	67 (9.2)	56 (9.1)	67 (8.3)	190 (8.9)
Retired *n* (%)	315 (43.3)	213 (34.7)	276 (34.3)	804 (37.5)
Other *n* (%)	252 (34.6)	218 (35.5)	293 (36.4)	763 (35.5)
Most deprived *n* (%)	509 (69.9)	429 (69.9)	552 (68.6)	1409 (69.4)
Least deprived *n* (%)	148 (20.3)	140 (22.8)	195 (24.2)	483 (22.5)
Rural *n* (%)	224 (34.1)	187 (32.8)	245 (32.9)	657 (33.3)
Urban *n* (%)	433 (65.9)	382 (67.1)	501 (67.1)	1316 (66.7)
Cancer stages *n* (%)				
Stage I	124 (29.5)	113 (26.9)	183 (43.6)	420 (19.6)
Stage II	128 (41.2)	75 (24.1)	108 (34.7)	311 (14.5)
Stage III	114 (37.8)	92 (30.5)	96 (31.8)	302 (14.1)
Stage IV	229 (30.4)	228 (30.3)	296 (39.3)	753 (35.1)
Unknown stage	133 (36.8)	106 (28.6)	122 (33.8)	361 (16.8)

“Due to some instances of missing data, cells may not add up to total number”

### Annual percent change (APC)

**Fig. 1:**
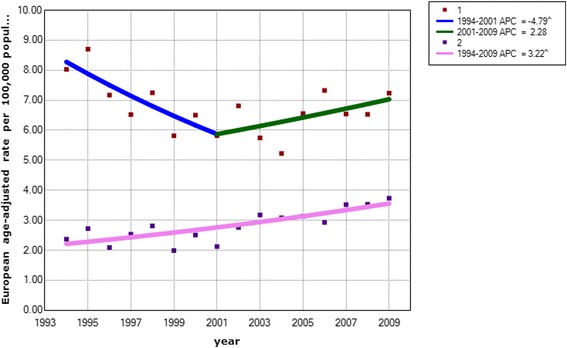
Estimated annual percent in oral cancer incidence among males (*top*) and females (*bottom*), 1999-2009, Ireland: 1: 1 Jointpoint versus 2: 0 Joinpoints

In females, oral cancer incidence rose significantly, by 3.2 % per annum (95 % CI: 1.9 to 4.6), during 1994–2009 (Table 2/Fig. 1). In contrast, an annual decline of −4.8 % (95 % CI: −8.7 to −0.7) was observed for male OC in the period between 1994–2001. In the most recent 8 year interval (2001–2009), the trend for men was judged to be stable i.e. a non-significant change at 2.3 % (95 % CI: −0.9 to 5.6) (Table 2/Fig. 1).

**Table 2:** Estimated annual percent change of oral cancer incidence 1994–2009, in Ireland

Sex	Year	Estimated annual percent change	95 % confidence interval
Female	1994–2009	3.2^a^	1.9, 4.6
Male	1994–2001	−4.8^a^	−8.7, -0.7
	2001–2009	2.3	−0.9, 5.6
Total	1994–1999	−5.2^a^	−10.0, 0.2
	1999–2009	2.6^a^	0.8, 4.3

^a^The estimated annual percent change (APC) is significantly different from zero at alpha =0.05

### Lifetime cumulative risk incidence

The lifetime cumulative risk of incidence from OC to age 79 years for the period between 2007 and 2009 was estimated to be 0.7 % and 0.5 % for males and females respectively. In other words, on average, seven men out of 1000 and five women out of 1000 have a current risk of being diagnosed with OC.

### Multivariable analysis

A multivariable Cox regression model assessed the risk of oral cancer adjusted for all potential confounders available in the study (Table 3).

**Table 3:** Multivariable Cox proportional hazards regression analysis

Variables	Hazard ratio (95 % CI)	*P*-value
Sex (reference = women)		
Men	1.1 (0.9–1.3)	0.15
Age (years) (reference = 30–60)		
< 30	0.3 (0.1–0.9)	0.03
> 60	1.6 (1.4–1.9)	<0.001
Smoking status (reference = non-smoker)		
Current	1.3 (1.1–1.5)	0.003
Former smoker	1.0 (0.8–1.3)	0.8
Unknown	1.5 (1.2–1.8)	0.001
Marital status (reference = married)		
Divorced	1.7 (1.1–2.7)	0.02
Single	1.2 (1.1–1.4)	0.002
Other	1.3 (1.2–1.5)	<0.001
Occupation status (reference = employed)		
Unemployed	1.3 (1.1–1.6)	0.003
Retired	1.5 (1.3–1.9)	<0.001
Other	1.1 (0.9–1.4)	0.18
Deprivation index (reference = most deprived)		
Less deprived	0.9 (0.8–0.9)	0.03
Years of incidence (reference = 1994–1999)		
2000–2004	0.8 (0.7–0.9)	0.01
2005–2009	0.7 (0.6–0.8)	<0.001
Tumour site (reference = C03 Gum)		
C01Base of the tongue	1.3 (1.1–1.7)	0.01
C02 Tongue	1.3 (1.0–1.6)	0.04
C04 Floor of mouth	1.2 (0.9–1.5)	0.11
C05 Unspecified mouth	1.1 (0.5–0.8)	0.62
C06 Palate	1.1 (0.8–1.4)	0.55
Cancer stages (reference = Stage I)		
Stage II	1.4 (1.1–1.7)	0.004
Stage III	1.8 (1.5–2.3)	<0.001
Stage IV	2.8 (2.3–3.3)	<0.001
Unknown stage	1.7 (1.4–2.1)	<0.001

From the Cox regression, being of older age (HR 1.6, *P* < 0.001), being a current smoker (HR 1.3, *p* = 0.003) and having a cancer at the base of tongue were all strongly associated with death. Conversely, being married, having a recent diagnosis of cancer (2005–2009), being less deprived and being employed were negatively associated with risk of death from oral cancer. There was a linear relationship between cancer stage and risk of death.

## Discussion

Oral cancer is a significant health problem, with changing patterns in many countries including Ireland, suggesting underlying changing lifestyle factors or putative risk factor changes.

From this retrospective study of all OC cases nationally in Ireland between 1994 and 2009 we found that the increase in incidence for women was driving the population level estimate. There was a significant annual increase of 3.2 % in OC among women from 1994 to 2009 similar to previous studies both in Ireland and elsewhere [28, 29].

In contrast, the trend for male OC incidence rates changed markedly between 1994 and 2009. A significant annual decrease in incidence of 4.8 % between 1994 and 2001 and a non-significant increase of 2.3 % was observed for the remaining years 2002–2009.

The lifetime incidence risk of OC for males was 0.7 % compared to 0.5 % in females between 2007 and 2009. Our estimates broadly agree with European estimates for females [6]. In 2004, the estimated lifetime risk of developing OC was 0.37 % for females in European Union countries. Our analyses show that men living in Ireland are at a lower risk than their European counterparts who had a risk of 1.85 % in 2004.

Specific survival rates in OC also varied in the study population. Smokers (HR 1.3), older patients (HR 1.6), more deprived patients and patients in advanced stages of OC cancer in which the tumour has extended beyond the organ or site of origin had significantly poorer survival rates. This result is consistent with the Neighbourhood Deprivation study in the United States [30],which reported a decreased survival rate amongst the most deprived patients between 1996 and 2009. An important factor in this context is the effect of the economic crisis in 2008. The continued decline in overall survival among the most deprived might have been compounded by the economic recession in 2008 when the Irish health care system was deeply impacted [31, 32]. However, the impact of a recession on health generally displays a significant time lag, so the whole picture will only be visible over the long term [33].

We also found that patients who developed cancers at the tongue and base of the tongue had lower survival rates than patients with other types of cancers. This might be due to asymptomatic presentation of most patients with tongue cancer leading to more advanced cancer on detection or misdiagnosis of the tumour by the attending clinician, in addition to the early nodal spread in this form of cancer [34].

Some of the observed trends may be related to underlying patterns of tobacco consumption among men and women over the past decades. While there has been a decrease in tobacco use across Europe over the last ten years, the consumption has decreased at a slower rate in women [35]. Regardless of the introduction of powerful measures like a workplace smoking ban in 2004, the average smoking rates in Ireland remained high at 29 % in 2007 [12]. In 2006, the Health Behaviour in School Aged Children survey reported that smoking was higher amongst girls than boys for children aged 12–14 years and 15–17 years [36]. Smoking was also more common among lower social class groups in the Health Service Executive (HSE) survey 2007 [36].

The survival rate of OC was significantly increased amongst married patients. Similar results were reported by Schaefer et al. in a study of 9403 elderly patients with oral cavity and pharyngeal cancers in the United States [37]. Married patients have better health behaviours such as medication adherence, and are subject to encouragement by spouses to seek medical care for worrying signs [38]. Psychologically, after a cancer diagnosis, married patients display less anxiety and depression than their unmarried counterparts, as a partner can provide social support and share the emotional burden [39]. This phenomenon raises the possibility of unmarried patients being a suitable target for social support interventions that may improve survival.

In our study, the survival of OC decreased progressively with age. Better prognosis for the recently diagnosed OC rates may be associated with some time-dependent factors related to changing behaviour and/or environmental exposure such as alcohol/tobacco consumption and HPV vaccination, which unfortunately we could not control for in our analyses. The improvement in procedures of diagnosis, early referral, and treatment options of oral cancer over the years may also be contributory [40]. The Mouth Cancer Awareness Day in Ireland is a good example of a good public health intervention in this regard, albeit this was introduced after the end of our study period. The national campaign commenced in 2010 and offers free dental examinations for anyone who wishes to be examined, with the aim of early detection of oral cancer [41]. The campaign was successful; discovering an additional 22 new oral cancer cases between 2010 and 2013 [42].

Other health promotion approaches in Ireland that impact on oral health include the three-year alcohol awareness campaign “less is more” that was implemented by the Health Promotion Unit (2001–2003). The aim of this programme was to increase awareness and initiate consideration of health and societal related alcohol issues [43].

Initiatives such as these have been shown in other countries to have an impact on oral cancer incidence rates [44]. The joint effect of alcohol and smoking consumption have been shown to be strongly associated with OC [7, 8, 45]. Although we did not allow for their combined influence in our models due to an absence of data on alcohol consumption, this is a further point for research.

## Strengths and limitations

We used a population-based study, using all cases of oral cancer in Ireland, to examine oral cancer incidence between 1994 and 2009. We used a comprehensive nationally representative individual-level oral cancer dataset with >97 % coverage. The advantage of employing joinpoint analysis, which has been consistently applied by the NCI in the United States, allows identification of significant inflexions in trend data. We have also estimated lifetime incidence risk of OC. Such an analysis might indicate underlying patterns of etiological importance that are amenable to both prevention and control of oral cancer burden in Ireland.

Robust multivariable modelling was performed to adjust for potential confounders available to the study.

However, there are study limitations. Information on important risk factors such as oral HPV status, data on oral hygiene and intake of fruit and vegetables were not available in our study. Additionally, we could not study the combined effect of alcohol consumption and smoking due to a lack of data on alcohol. These data limitations may have resulted in some residual confounding.

## Conclusion

In conclusion, while the incidence of oral cancer increased significantly in both sexes between 1999 and 2009, the overall population increase appears to be driven by women (APC 3.2 % per year). From our analyses, survival was better for younger and affluent groups compared to older and deprived patients. Survival rates of OC were also higher in early cases and in married couples.

Although in this study we could not control for every single risk factor, the existing evidence in combination with our contribution points to alcohol use, tobacco, dietary factors, HPV, social class in addition to time of diagnosis and age as all being important factors in the causation and prognosis of OC. Many of these factors are modifiable and thus should be emphasized as major targets for public policy aimed to positively impact the numbers of oral cancer.

## Abbreviations

APC: Annual percent change
CI: Confidence intervals
ED: Electoral division
HPV: Human papillomavirus
HR: Hazard ratios
HSE: Health service executive
ICD: International Classification of diseases
KP: Kaplan-Meier
NCRI: National Cancer Registry Ireland
OC: Oral cancer
OECD: Organisation for economic co-operation and development
